# Application of the COM–B Framework to Understand Facilitators and Barriers for Practising Physical Activity among Pregnant Women and Midwives Participating in the WELL-DONE! Study

**DOI:** 10.3390/bs13020114

**Published:** 2023-01-30

**Authors:** Sofia Marini, Rossella Messina, Alice Masini, Francesca Scognamiglio, Isotta Caravita, Vincenza Leccese, Giorgia Soldà, Dila Parma, Virginia Bertini, Lawrence Matthew Scheier, Laura Dallolio

**Affiliations:** 1 Department for Life Quality Studies, University of Bologna, 47921 Rimini, Italy; 2 Department of Biomedical and Neuromotor Science, University of Bologna, Visa San Giacomo 12, 40126 Bologna, Italy; 3 Department of Medical and Surgical Sciences, University of Bologna, Bologna Via Massarenti 9, 40138 Bologna, Italy; 4 LARS Research Institute, Inc., Sun City, AZ 85351, USA; 5 Prevention Strategies, Greensboro, NC 27410, USA

**Keywords:** physical activity, pregnancy, health promotion intervention, feasibility, focus group method, COM–B model, midwives

## Abstract

Regular physical activity (PA) is protective and reduces disease burden but remains a challenge for pregnant women (PW). According to the World Health Organization (WHO) guidelines, PW without contraindications should practice 150 min of moderate PA per week. Nonetheless, PA levels are concerningly low among PW. The aim of this study was to investigate PW’s and midwives’ perceptions regarding PA and recommended guidelines, and use this information to inform future health promotion strategies. We recruited 10 PW and 10 midwives to participate in online focus groups conducted between July 2020 and April 2021. Focus group probes and data analysis were guided by the COM–B (capability, opportunity, motivation–behaviour) framework. The majority of the sample had already practised PA, recognized the importance of PA during pregnancy, and considered the WHO guidelines reasonable. Notwithstanding, PW wanted more specific instruction on PA and desired opportunities to practice. Additional barriers reported by PW included low self-efficacy and lack of motivation. Midwives considered the lack of specific knowledge and confidence in managing PA as the main obstacles. The current findings suggest that PW and midwives need specific training in PA to overcome both psychological and physical barriers. Midwives play a vital role in educating and encouraging PA among PW.

## 1. Introduction

Physical activity (PA) is a key lifestyle factor with tremendous implications for health prevention and health promotion. Indeed, evidence shows that low levels of PA and sedentary behaviour correlate with several poor health outcomes at all stages of life. Conversely, individuals with an active lifestyle benefit in many ways, including better physical and mental health [1,2,3]. Physical activity is especially important during pregnancy, where regular PA is associated with favourable maternal and foetal health outcomes (i.e., a decreased risk of pre-eclampsia, gestational hypertension, gestational diabetes, excessive gestational weight gain, delivery complications, postpartum depression and newborn complications) [4,5,6,7,8]. Indeed, PA can modulate several physiological changes that occur during pregnancy (i.e., haemodynamic changes such as increased blood volume, plasma and red blood cell mass, heart rate changes) and act on a variety of biological pathways (e.g., neurotransmitter release), thus favouring optimal maternal outcomes in terms of physical and mental health (e.g., better sleep quality, less depression, anxiety and stress) [9,10].

Based on accumulation of evidence favouring positive outcomes with PA, the World Health Organization (WHO) established guidelines that all pregnant women (PW) should engage in regular PA [11]. The guidelines suggest performing 150 min of moderate physical activity weekly, in order to achieve sustainable health benefits [11]. Moreover, the same guidelines also specify that including aerobic and muscle strengthening activities can enable PW to obtain physical and psychological benefits [11]. Notwithstanding, PA levels in PW tend to be generally extremely low [12], with only a reported 15% of this population closely adhering to the WHO guidelines [11].

From a public health perspective, several strategies can be adopted to tackle sedentary lifestyles and improve PA levels in PW. Health promotion interventions delivering adapted physical activity programs (APAs) to PW have proven to be effective strategies that can enhance PA levels in this population [13]. Different types of interventions can be delivered, depending on the pre-pregnancy PA levels of PW and also considering their health status. Moreover, interventions of this nature should take into account local context (e.g., cultural, structural, environmental) as well as consider possible barriers and facilitators that may mitigate the effectiveness of PA-based interventions. Unfortunately, there is a paucity of information revealing the nature and quality of barriers and facilitators in PW and how this may affect their involvement in PA. Qualitative methods, such as focus groups, provide one means of obtaining this information and learning more about barriers and facilitators. Focus groups are especially useful because they can provide researchers with a more in-depth, if not nuanced, understanding of the experiences, motivations, and perceptions that PW have toward PA. This information can then be utilised to design and implement effective programs with the target population in mind [14].

The basic format for focus groups involves small group discussions guided by a moderator/facilitator. The moderator has experience running small groups and is adept at managing group dynamics. The moderator relies on “probes” or semi-structured questions to elicit discussion by the participants in a free-flowing manner. Although participants individually answer the facilitator’s questions, they are usually encouraged to bounce ideas off each other and engage in social discourse [15,16,17,18,19,20].

Consolidating the various themes that run through a focus group discussion requires some type of overarching framework [20]. This is helpful not only as the group discussion unfolds, but also to organise the myriad of comments resulting from transcription of the focus group conversation. In the present study, we used the COM–B model proposed by Michie et al. [21]. As a whole, COM–B subsumes theories of human motivation and agency that highlight the role of cognition (reflective thought processes), emotions and habitual processes. The COM–B can be conceived of as a system of interrelated dynamic processes that can assist program developers to identify proper targets for healthy lifestyle interventions (i.e., the core components or active ingredients of the intervention) and the best way to achieve behaviour change (i.e., specific intervention functions to include in a particular program).

The COM–B framework is often depicted within a series of concentric circles forming a behaviour change wheel (BCW). The most interior portion of the wheel, considered the “hub,” represents the necessary conditions for a behaviour to occur. It consists of the following three interacting dynamic systems: capability, opportunity and motivation. Capability to perform a specific action depends on mechanisms that are internal to the individual (i.e., knowledge and skills) and reflects their physical (i.e., musculoskeletal strength) and psychological (i.e., thought processes and reasoning) capacity. It is the level that addresses personal agency within the control of the individual. Opportunity refers to external factors that can facilitate behaviour. It is the contextual level that addresses the sociocultural milieu, including resources and supports. Motivation reflects a combination of both conscious (analytical and reflective decision making) and unconscious (habitual fast-acting processes like impulses and emotion-based decision-making) mental processes that lead the individual to perform a behaviour [21]. To illustrate its utility, consider, for example, a situation in which PW all identify their weight during pregnancy, lack of muscular strength and fatigue as stopgaps that prevent them from engaging in PA. A program developer can then incorporate this information into a health promotion setting teaching body balance, muscular tone and healthy nutritional practices, in order to offset lifestyle changes that may result from pregnancy.

The next concentric ring in the BCW represents the actual intervention functions and can include a variety of strategies (e.g., persuasion, training, education, restrictions, coercion, as a few examples of the nine intervention functions). These represent the modalities that enact behaviour change by addressing deficits in the COM–B hub. The outer rim of the wheel represents policy and advocacy that can be used to support intervention delivery [21]. This would include fiscal policy (e.g., taxing cigarettes), regulatory change (e.g., drunk driver laws), health promotion guidelines, service provision, communication/marketing and environmental/social planning efforts that are implemented with the goal of intervention development and behaviour change.

In its entirety, the BCW, can be thought of as a step-by-step guide for program designers and implementers that helps them identify and address deficits in capability, opportunity and motivation through specific behaviour change, policy and advocacy strategies.

It is with this in mind that the WELL-DONE study! [22] was designed to improve PA in PW. Prior to introducing the intervention, and guided by the BCW, we used focus groups to glean information that could improve the design, implementation, and evaluation of the intervention. From a logic model perspective, the goal was to create the best fit possible, given any implementation constraints, learn more about potential routes for diffusion of the intervention (in hospital and clinic settings) and ensure its sustainability over time. Outcomes included (1) PW’s attitudes towards the practice of PA during pregnancy, (2) obstacles and solutions to the promotion and practice of PA and (3) identifying the intervention components to consider when designing PA within childbirth preparation classes (CPCs).

## 2. Materials and Methods

Six 90 min long focus groups involving PW and MWs were carried out between 2020 and 2021 at the Sant’Orsola University Hospital, located in Bologna, Italy. The study was approved by the Ethics Review Committee for the Emilia–Romagna Region, Italy, on the 11 November 2020 (Prot. n. 984/2020/Sper/AOUBo of 19/11/2020).

The study followed the Standards for Reporting Qualitative Research (SRQR) checklist. Invitation letters were sent both to MWs working at the Operative Union of Obstetrics at the University Hospital and to PW who planned to attend the CPCs organised by the hospital. PW who wished to participate in the study (i.e., focus groups) were contacted via telephone. During this initial contact, members of the research team described the study requirements. Including MWs at this formative stage is crucial as they play a key role in offering support, education and training to women during pregnancy. As a result of their integral role in the birthing process, they are cognizant of the full range of potential issues PW may encounter during their pregnancy including reasons for their reluctance to incorporate PA in their daily regimen.

Written consent was obtained immediately prior to participation in the focus groups. There were no exclusion and inclusion criteria for participating in the focus groups. Focus groups were held online using multimedia communication platforms (Zoom^®^ and Microsoft Teams^®^) as the study timeframe occurred during the COVID-19 pandemic. Focus groups were video- and audio-recorded and data were transcribed verbatim, which provides researchers with a chance to revisit the content of the conversation. The transcripts and supplemental notes written by moderators and observers were coded and analysed using a grounded theory approach and based on the COM–B model [23].

Moderators initiated each focus group with an ‘icebreaker’ activity to familiarise participants with the setting (e.g., how PW feel toward health promotion or PA) and explain rules governing group behaviour (i.e., participation was confidential, respecting each other’s opinions, not interrupting and using first names only). This could be followed by more specific questions (e.g., things that prevent PW from exercising) that build off the initial set of probes. Each focus group lasted two hours and was divided in four main themes. During the meeting all participants were invited to think about the questions shown by the moderator and shared their personal opinions and beliefs related to each question in all the themes. We held separate focus groups, first with MW, then with PW to ask them the same questions, based on their different points. As the conversation among participants takes shape, the moderator promotes a more in-depth discussion on relevant topics to further elucidate the participants’ sentiments on the topic. This helps the group drill down deeper into the subject matter and, at the same time, provides a means for the researchers to gain perspective on the desires, wishes and needs of the focus group members.

The introduction involved a series of general questions (i.e., number of pregnancies, previous experience with PA, pros and cons of PA during pregnancy). This phase was followed by the presentation of the WHO guidelines and a brief discussion regarding their feasibility. Barriers and facilitators were discussed and divided into four main categories (mother-related, baby-related, childbirth-related and social context-related factors). The last part of the focus groups was used to collect participants’ opinions about delivering a PA-based intervention during CPCs.

## 3. Procedures and Data Analysis

As one of several qualitative techniques, focus groups strike a chord as a “middle” ground between intensive stakeholder interviews that provide in-depth material obtained from a single individual and participatory research, the latter of which relies solely on naturalistic observation with no formal contact between observer and subject. The material obtained from focus groups is then subject to analysis using the principles of grounded theory, with an emphasis on discovering recurrent and prominent themes that resonate during the participants’ discussion. Focus group probes were developed in collaboration with a psychologist (RM). Two moderators managed the focus group and the groups were attended also by two (silent) observers. Moderators facilitated participants’ interactions and used the probes to develop discussion based on the COM–B model. The aim was to specifically investigate what facilitated or hindered PW engaging in PA. Both moderators (and the observers) took notes and the data were transcribed verbatim. The observers took notes on participants’ reactions and expressions and monitored the group dynamic. Moreover, two Public Health medical residents from the University of Bologna also (silently) observed the focus groups and took copious notes for each group. The transcripts and notes were imported into NVivo (V.11). Data analyses were conducted using a seven stage approach, as follows: data familiarisation, thematic coding using the COM–B model, identification of sub-themes within the framework, review and revision of subthemes, definition and naming of sub-themes, analysis and interpretation of patterns across the data and amalgamation of sub-themes into dominant contextual domains. The interview transcripts were coded and initially analysed by two independent researchers, and then a third person resolved any ties to reach consensus.

## 4. Results

### 4.1. Sample Characteristics

The mean age of PW attending the focus groups was 34.10 ± 4.46 years, with 80% reporting they were primiparous. All PW had practised PA before pregnancy;, involving a variety of sporting endeavours; with tennis, jogging, yoga, swimming, dancing, gymnastics, exercising in the gym, martial arts and fast-paced walking being the most frequently reported activities. The mean age of the MWs was 53.80 ± 5.49 years, 90% of them were primiparous and only 50% of them reported having previous PA experience.

### 4.2. Barriers and Facilitators for Practising PA

Table 1 provides an overview of the main concepts that emerged during the group discussion and that agree with the COM–B framework. PW reported lack of specific knowledge about PA during pregnancy and pregnancy-related factors (e.g., tiredness during the first and third trimester, having a high-risk pregnancy) as the main barriers to performing PA. Pregnant women also reported work-related issues, family issues and having a partner with a sedentary lifestyle as obstacles to PA. Finally, negative social views towards PA during pregnancy by significant others (i.e., injunctive norms), not being motivated to perform PA alone, and fear of hurting the baby were important motivational factors that prevented PW from engaging in PA.

“It is difficult for me to find time to dedicate to physical activity”...(PW)

“I am scared to do wrong movements that can hurt my baby”...(PW)

“I received many tips from others that have hindered me from exercising (i.e., mother, mother-in-law, partner...), they are not required and make me feel anxious even if I am aware of what I can and what I cannot do”...(PW)

Midwives identified similar barriers they felt inhibited PW from engaging in PA practice. In addition, MWs underlined the importance of cultural factors (e.g., some PWs have a cultural background that does not favour connection with bodily movement, have a sedentary lifestyle, and do not frequently access CPCs). “Many pregnant women have sedentary lifestyles and low body awareness”...(MW)

Specific types of exercises were discussed during the focus groups (e.g., exercises to strengthen the perineum, training with a softball or other tools, water aerobic workouts). These kinds of exercises were considered instrumental in training PW to connect with their bodies, feel empowered and mentally prepared for the physical effort required during labour and delivery.

### 4.3. Co-Design Results

The final part of the focus groups involved obtaining feedback from both PW and MWs during a co-design phase to inform the development of the WELL-DONE protocol. When discussing how to structure the PA, the MWs suggested specific low-impact workout and bodyweight resistance exercises such as yoga and pilates. Likewise, they suggested activities focusing on postural and relaxing exercises (e.g., antalgic position, perineum exercises, birthing exercises and stretching). In addition to this feedback, PW reported the importance of including strengthening exercises within the protocol that would prepare them for the delivery phase and especially for postpartum recovery.

“It may well be important to include partner strategies for partners, within CPCs, to support the PW with movements and massages during labour”...(MW)

“I would like to address the PA topic during CPCs, receiving specific exercises that I can do during each period of pregnancy”...(PW)

Moreover, both MWs and PW reported the importance of including exercises that involved couples to create even more connection between partners. Both MWs and PW reported the importance of using music and performing exercise in a relaxed environment (e.g., warm rooms with soft ambient light). Midwives stressed the need for using play objects and materials in the PA sessions, for instance, using softballs or fitballs, as well as other support materials such as mats, tennis balls, or elastic bands. Finally, PWs suggested that the PA protocol should include information related to maintaining a healthy diet and learning about proper nutrition for PW and the foetus.

## 5. Discussion

Physical activity during pregnancy offers significant benefits in terms of maternal and foetal health. Nonetheless, only 15% of PW achieve PA levels recommended by the WHO guidelines (i.e., 150 min of moderate PA per week) [11]. Among several strategies that can be used to increase PA levels during pregnancy, PA interventions represent a valuable and cost-effective option. Pregnant women frequently visit their local medical clinic for routine maternal care checkups covering their health and that of the foetus. At these meetings, PW will work closely with MWs to track the course of their pregnancy, prepare them for delivery, and learn about postnatal care of the infant. It is also common for PW to form social bonds and band together with other PW and MWs during their pregnancy as a means of garnering social support, sharing insight and learning relaxation techniques that may help them during delivery. In light of this, it would seem relatively easy to develop opportunities for PW to engage in PA, even as part of group exercises, and capitalise on its health benefits. While an intervention of this nature may seem easy to design and implement, there are numerous obstacles that hinder such opportunities, making it a relatively difficult task to accomplish in a real-world setting. Indeed, a wide range of barriers and obstacles have been noted in the literature that mitigate the value of health promotion programs with PW [24].

To address these concerns, we set out to learn more about what factors might inhibit PW from engaging in PA as well as factors that might facilitate developing and implementing a PA-based intervention. We extended this focus to include MWs as they play a vital role in the education of PW and can have substantial influence on their engagement in PA, thus being an ideal implementer of this type of intervention. Indeed, the intervention setting would involve PA training sessions that could be delivered during CPCs held in local hospitals. This type of setting represents a good fit because it readily addresses sustainability (MWs specifically trained by kinesiologists can deliver the intervention without the need to hire a specific professional to fulfill this task) and equity (CPCs in Emilia–Romagna, a region in the north of Italy, can be accessed by all PW for free). To better understand how to structure PA for these women, we used focus groups providing a platform for PW and MWs to participate in co-designing an intervention from the ground up. Focus groups provide researchers with a unique opportunity to gain an in-depth knowledge of a particular topic of investigation directly involving the target population (PW) or other significant figures, such as in our case involving MWs. It is an open-ended methodology, during which participants can freely discuss their feelings toward a specific topic (i.e., PA practice during pregnancy). In the current study, PW used the opportunity to address what holds them back, express their innermost fears about pregnancy and physical exercise and discuss what would facilitate their participation in PA (e.g., whether they feel their partner would support them and provide assistance when needed). Midwives disclosed their unique perspective about factors influencing PW to take up PA, and also the barriers that they perceive as professionals (i.e., what prevents them from offering adequate guidance and support to PW during PA practice).

Overall, all participants shared similar views about PA during pregnancy and they identified similar barriers and facilitators to PA practice. A core element emerging from the focus groups was the participants’ emphasis on knowledge (psychological capability) and skills (physical capability). Without adequate information about PA during pregnancy (e.g., PW not knowing that PA is safe and provides broad maternal–foetal health benefits) and specifically about exercises and movements that can be practised in this particular period, PW voiced that they feel insecure and therefore less motivated to engage in PA. On the other hand, MWs felt they lacked adequate training about PA and therefore were incapable of supporting PW during PA practice. The lack of skills and education on the benefits of PA during pregnancy contributed to doubt and concern on their part. Accordingly, high quality training for MWs should enable them to deliver a personalised PA intervention based on PW’s pre-pregnancy knowledge and skills. A focus on capability (knowledge and skills) emerged in both groups as vital to help them overcome other types of perceived barriers (e.g., social and motivational). Both midwives and PW reported that correct information provided by qualified healthcare professionals can help them gain a more informed perspective and counteract social pressures hindering PA practice (e.g., negative views towards PA expressed by family or by their partner). Lack of social support was indeed another critical element reported by all participants. In particular, MWs stressed the impact of cultural factors (e.g., attitudes towards women’s bodies and bodily functions during pregnancy) on PW’s attitudes towards PA, while PW underlined the importance of involving their partner and other children in PA practice. From this perspective, structured communication could serve as an important means to address fears and doubts regarding PA, increase social support and motivate PW to engage in PA.

### 5.1. Theoretical Implications

The COM–B model provided a conceptual framework to organise construction of the focus group probes, as well as investigate (and code) the open-ended textual discourse among the PW and MWs. In the simplest sense, COM–B provides a framework to translate desired outcomes (behaviour) into mechanisms of action. Toward this goal, COM–B elucidates the following three facets of behaviour that inform the basis of an intervention: capability, opportunity and motivation. Using this framework, COM–B then defines targets for intervention and sets specific behavioural change goals. COM–B also provides a necessary linkage with intervention strategies’ functions, as it is embedded in the larger “behaviour change wheel,” which characterises interventions in terms of the behavioural target and specific intervention function. When interventions “fail,” at the very least, the implementation team can identify which active elements were targeted and in terms of which specific desired behavioural outcomes.

In the present context, participants identified barriers related to all the components of the COM–B model, thus supporting its comprehensiveness and utility as a framework. Primary targets identified through focus groups included lack of physical and psychological capability, social and physical opportunities, as well as (and to a lesser degree) reflective and automatic motivation. Overall, these barriers should represent the “targets” of an effective PA intervention earmarked for PW. The sheer fact that so many targets were identified as potential barriers to address suggests that a multimodal intervention will be best suited for the target audience. From an implementation standpoint, the following three factors stand out as foci for intervention development: the need for greater training, education and environmental restructuring to help PW achieve their PA goals. Training (imparting new skills) and education (increasing knowledge) can address lack of physical and psychological capabilities and empower PW with a greater sense of confidence to execute the required PA exercises using their newly acquired skills. On these grounds, a strong educational component needs to be introduced in order to improve body awareness and enable PW to perform PA independently. Moreover, PA protocols should be designed to take into consideration pre-pregnancy PA levels and, therefore, personalising PA based on PW’s health status (construction of a strong motor–sensory background for sedentary women and maintenance of previous PA levels for active women). For PW who have not engaged in PA, this could include muscle tone and balance through stretching, core stability exercises, yoga classes and cardiorespiratory exercise routines (e.g., treadmill walking) that maximise VO_2max_ (aerobic exercise training), all of which can be delivered in brief training sessions to build confidence. For more active women, this can include similar training delivered over more extended time frames with personalised goals and weekly charts to monitor increases in activity (e.g., pedometer readings), supplemented with weight and non-weight bearing exercise routines (e.g., cycling and water aerobics), calorie intake and nutrition charts, the latter to address increased metabolic and physiological demands of pregnancy (i.e., offsetting hypoglycaemia from moderate exercise through increased calorie intake and offsetting hyperthermia through proper hydration).

### 5.2. Physical Activity Training

Midwives can also benefit from specific training in PA by supplementing their education with respect to exercise physiology and muscle tone fitness exercise routines. Midwives are frequently the main point of contact for PW in the healthcare system and, as a result, they can build trust over time. Midwives will likely be present through the duration of the pregnancy and sometimes even remain in contact with PW postpartum. This continued presence affords them a terrific opportunity to use their knowledge to inform PW regarding the positive benefits of PA during pregnancy. Midwives can receive expert training from kinesiologists, with the specific goal of providing education on pregnancy-related changes to the body, musculoskeletal movement to alleviate organ and valvular compression and cardiovascular benefits of exercise to the PW and foetus. In this way, MWs will feel more competent and enabled to provide specific instructions and advice regarding PA at CPCs, as well as monitor PW during PA practice. Along with highly trained professionals to allay concerns over harming the foetus, training sessions can address breath control, pain management and motor control, with particular attention paid to the pelvic floor with specific exercises. This training can help reduce barriers preventing PW from performing PA and enhance positive feelings associated with PA practice.

Another area that might be fruitful for encouraging PA involves environmental restructuring that could help address several physical and social barriers reported in the focus groups (e.g., providing an area where PW can put their other children while attending the CPCs). Providing childcare, food and activities might encourage women with larger families to bring their other children and make PA a “family outing.” Involving other children in the family or a partner in PA practice can increase social support and ease family-related issues. The same holds for encouraging sedentary partners, who could utilise the space for exercise and learn how social support enhances pregnancy outcomes (i.e., improves well-being in the PW). For these reasons, partners should also be involved in CPC sessions, in order to disseminate information about PA importance during pregnancy and help partners realise their instrumental importance for continued health and well-being of the PW and foetus. A personalised and flexible schedule can also be a possible solution to time-related, work-related and family-related barriers. Organising brief sessions that could fit in the daily routine and distributing PA during the week can help overcome lack of time, as well as walking to (or at) work and limiting sedentary behaviour (following the rule “every move counts”). Rarely are people fully aware of their attitudes toward engaging in a behaviour (i.e., sedentary vs. active lifestyles) or why they think in a particular way toward behaviour. Pregnant women may have certain preconceived notions about PA during pregnancy, but are not aware that these ideas limit their engaging in a particular behaviour.

### 5.3. Study Limitations

This study involves several limitations worth noting. The focus groups were based on a small sample of PW and MWs, which may not reflect the broader feelings of women giving birth and their healthcare support network. The project commenced during the COVID-19 pandemic, making it challenging to recruit participation from both PW and MWs. We also did not involve any members of the PWs’ support groups, including significant others and spouses. More often than not, these individuals provide psychological support during pregnancy. The absence of this support can hinder participation in exercise classes, as much as its presence can bolster support. In addition to significant others, healthcare professionals in the medical community can also be instrumental in promoting PA among PW. This can extend beyond the MWs, who are the “face” of obstetrical and prenatal care in many places. This is particularly true of low-income countries and places where travel to main hospital centers is cumbersome. Including a wider range of individuals involved in healthcare and support networks can only serve to reveal additional factors that may inhibit or promote exercise in PW as well as identify how to overcome any barriers to obtaining better maternal healthcare.

Theoretically speaking, the COM–B model provides a useful framework from which to interpret the focus group discussions. If there is a weakness in the framework, it lies in what is considered reflexive, automatic cognition (i.e., beneath the radar of consciousness). This suggests that less is known about which strategies could underpin ‘motivation,’ compared to what is known about conscious, reflective thought that involves direct skill training. Like many other intervention–implementation frameworks, COM–B does not elucidate how to approach intervention development (i.e., creating actionable items) when it comes to emotional content or other unconscious biases that might interfere with PW’s views toward PA. This type of implicit cognition is rarely examined in the context of the COM–B, perhaps owing to the different methodologies used to examine intervention-related behaviours in the context of an implicit paradigm [25].

### 5.4. Future Directions

Limited access to birthing or medical centers in low- and middle-income countries, and inability to obtain materials or props to facilitate exercise may prevent many PW maintaining a steady regimen of PA. Both geography and inclement weather can interfere with or hinder travel, and general fatigue linked to pregnancy may inhibit women from fully realizing the potential benefits of organized PA. Recent reviews and empirical studies confirm that, where connectivity permits, mobile phone health (mHealth) digital technology provides a cost-effective workaround to promote PA in PW [26]. This is especially true for individuals from low- and middle-income countries [27]. Moreover, early trial results showed that mHealth interventions can improve critical maternal ante- and prenatal health awareness and related behaviour in disadvantaged pregnant women [28,29,30]. Mobile and smartphone apps can be used as communication platforms to send personalized SMS text reminders (boosting adherence), maintain exercise calendars, track nutrition, answer questions and connect PW to medical professionals and bulletin boards where they can post concerns and receive real-time feedback. The technology also provides a means for PW to receive on-demand updates regarding their health status including monitoring vitals and results of accelerometer data that track PA and movement. There are opportunities for including self-help multimedia including videos to demonstrate exercise form. All of these technology capabilities can reinforce the importance of PA and strengthen the role of MWs who can interact through the smartphone app and provide positive emotional support (e.g., monitoring exercise progress and health status and responding to medical emergencies) to PW. This type of empowerment can go a long way toward sustaining PA in PW who might otherwise not take advantage of face-to-face, clinic-based exercise programs because they lack confidence, have anxiety, encounter barriers or do not feel supported.

## 6. Conclusions

The current findings suggest that PW and midwives need specific training in PA to overcome both psychological and physical barriers. Midwives play a vital role in educating and encouraging PA among PW. Delivering an effective PA intervention to PW can be challenging. Indeed, a variety of factors related to local context can have an impact on an intervention’s effectiveness. Qualitative methodologies such as focus groups provide an indispensable tool to investigate these barriers and strengthen the development and implementation of PA interventions through co-design activities. Complex information deriving from focus groups needs to be categorised using an overarching framework. In this respect, the COM–B model provides necessary but not sufficient conditions to design interventions that can address the full gamut of barriers and facilitators that underpin PW’s motivation to engage in PA. 

## Figures and Tables

**Table 1:** A. Barriers and facilitators for practising PA among pregnant women using COM–B framework. B. Barriers and facilitators to physical activity implementation among midwives across COM–B framework.

**A. Pregnant Women**			
**COM–B**	**Components**	**Barriers**	**Facilitators**
Capability	Physical	Pregnancy-related factors (i.e., pregnancy at risk, tiredness during the 1st and 3rd trimester)	Modulating intensity and duration of PA based on your daily health status; personalising PA based on pre-pregnancy PA levels
	Psychological	Lack of specific knowledge about PA during pregnancy	Healthcare experts should communicate clearly and transmit the importance of PA during pregnancy; provide PW with video tutorials and informative material (clear, simple, evidence-based)
Opportunity	Resources and supports	Work-related issues, lack of time, family issues (e.g., need to take care of other children)	Distributing PA during the week (i.e., do a small amount of PA every day); try and limit sedentary behaviour (i.e., walking as much as you can, take the steps and not the elevator); involving other child/children in the activities (e.g., going to the park together); organising brief PA sessions that could fit into the daily routine
		Lack of expert support and advice; having a partner with a sedentary lifestyle	Obtain expert support for personalised PA schedule; find a balance with partner’s habits and seek his/her support and involvement; talk with other PW who have already taken part in CPCs and PA programs
Motivation	Reflective	PA not perceived as a priority; social contacts’ negative views towards PA; not motivated to independently perform PA	Taking part in online classes or group activities (e.g., walking with other PW); vary exercise routine; perform PA in a friendly environment (e.g., relax by using music in a room with soft ambient lighting)
	Automatic	Fear of hurting the baby	Obtain reinforcement from expert that PA exercises are correct
**B. Midwives**			
**COM–B**	**Components**	**Barriers**	**Facilitators**
Capability	Physical	Pregnancy-related issues (tiredness, big belly during the 3rd trimester)	Demonstrating that PA can help overcome fatigue, tiredness and pain; provide personalised PA schedule based on PW conditions
	Psychological	Lack of knowledge about the importance of PA during pregnancy	Midwives and other professionals should explain exercises and provide sufficient time to practise movements
Opportunity	Physical	Work-related issues (lack of time, tiredness)	Walking to work
	Social	Cultural factors	Involve the partner; organising meetings with PW and their partner; organising meetings for PW outside CBCs
Motivation	Reflective	Influence of other people’s negative opinions towards PA during pregnancy	Midwives should listen to PW fears and explain importance and safety of PA during pregnancy
	Automatic	Fear of hurting the baby or inducing labour by practising PA, fear of pain	Midwives and other health professionals should explain the safety/importance of PA; present PA as self-care, opportunity to connect body with baby; present PA as a training tool for delivery

## Data Availability

Data are available by contacting the corresponding author.
